# Population genomics of divergence among extreme and intermediate color forms in a polymorphic insect

**DOI:** 10.1002/ece3.1928

**Published:** 2016-01-03

**Authors:** Jeffrey D. Lozier, Jason M. Jackson, Michael E. Dillon, James P. Strange

**Affiliations:** ^1^ Department of Biological Sciences University of Alabama Tuscaloosa Alabama; ^2^ Department of Zoology & Physiology and Program in Ecology University of Wyoming Laramie Wyoming; ^3^ USDA‐ARS Pollinating Insect Research Unit Utah State University Logan Utah

**Keywords:** Bumble bees, color pattern, divergence, gene flow, population genomics, RAD‐tags, RNAseq

## Abstract

Geographic variation in insect coloration is among the most intriguing examples of rapid phenotypic evolution and provides opportunities to study mechanisms of phenotypic change and diversification in closely related lineages. The bumble bee *Bombus bifarius* comprises two geographically disparate color groups characterized by red‐banded and black‐banded abdominal pigmentation, but with a range of spatially and phenotypically intermediate populations across western North America. Microsatellite analyses have revealed that *B. bifarius* in the USA are structured into two major groups concordant with geography and color pattern, but also suggest ongoing gene flow among regional populations. In this study, we better resolve the relationships among major color groups to better understand evolutionary mechanisms promoting and maintaining such polymorphism. We analyze >90,000 and >25,000 single‐nucleotide polymorphisms derived from transcriptome (RNAseq) and double digest restriction site associated DNA sequencing (ddRAD), respectively, in representative samples from spatial and color pattern extremes in *B. bifarius* as well as phenotypic and geographic intermediates. Both ddRAD and RNAseq data illustrate substantial genome‐wide differentiation of the red‐banded (eastern) color form from both black‐banded (western) and intermediate (central) phenotypes and negligible differentiation among the latter populations, with no obvious admixture among bees from the two major lineages. Results thus indicate much stronger background differentiation among *B. bifarius* lineages than expected, highlighting potential challenges for revealing loci underlying color polymorphism from population genetic data alone. These findings will have significance for resolving taxonomic confusion in this species and in future efforts to investigate color‐pattern evolution in *B. bifarius* and other polymorphic bumble bee species.

## Introduction

The parallels between phenotype and lineage diversification, and the underlying genetic mechanisms of rapid phenotypic change during divergence, are topics that continue to receive considerable attention in evolutionary biology (Schluter 2001; Gillespie 2004; Nosil 2009; Nadeau and Jiggins 2010; Devitt et al. 2011; Conte et al. 2012; Kronforst and Papa 2015). Geographic variation in insect coloration, particularly with respect to mimicry, has provided some of the most intriguing examples of the ecological and evolutionary drivers of phenotypic divergence and convergence among closely related populations and species (Bates 1862; Müller 1879; Williams 2007; Kronforst and Papa 2015). These systems can be especially useful as a means of elucidating the roles of genetic isolation and exchange among lineages (Mallet and Joron 1999; Nosil 2009; Hines et al. 2011; *Heliconius* Genome Consortium 2012), both of which may be important for the distribution of adaptive variation. For example, divergent phenotypes may stem from accumulation of unique genetic variants among populations due to reproductive barriers, drift, and selective pressures that maintain differentiation in distinct environments (Nosil et al. 2005). Conversely, the spread of beneficial alleles through hybridization and selection may contribute adaptive phenotypic evolution across species (Seehausen 2004; Gompert et al. 2006; Mallet 2007; Hines et al. 2011). Understanding the relative contributions of divergence and admixture in populations that are in the process of diversifying may help reveal the evolutionary forces at play in the origins of phenotypic variation like coloration.

Bumble bees commonly exhibit extraordinary color‐pattern variation (Plowright and Owen 1980; Williams 2007) that provides opportunities for investigating the interaction of phenotypic polymorphism and diversification in closely related lineages. Convergence of sympatric species on similar pigmentation patterns has produced more than 30 “Müllerian mimicry complexes” globally (Plowright and Owen 1980; Williams 2007). Intraspecific populations in different geographic regions may evolve different color patterns to match local mimicry complexes, while multiple species in the same region phenotypically converge (Williams 2007; Owen et al. 2010; Hines and Williams 2012), and untangling the relationships between genetic divergence and phenotype can thus be a challenge. Indeed, associations between phylogeny and color at deeper timescales are often weak (Cameron et al. 2007; Hines and Williams 2012), suggesting rapid evolution of pigmentation changes that might be attributed to the relatively small set of “color‐pattern elements” (Rapti et al. 2014) and associated genes. However, lineage associations with color are found at lower phylogenetic levels (Duennes et al. 2012; Hines and Williams 2012; Lozier et al. 2013), which may ultimately be most useful for illuminating the processes involved in pigmentation evolution.

**Figure 1:**
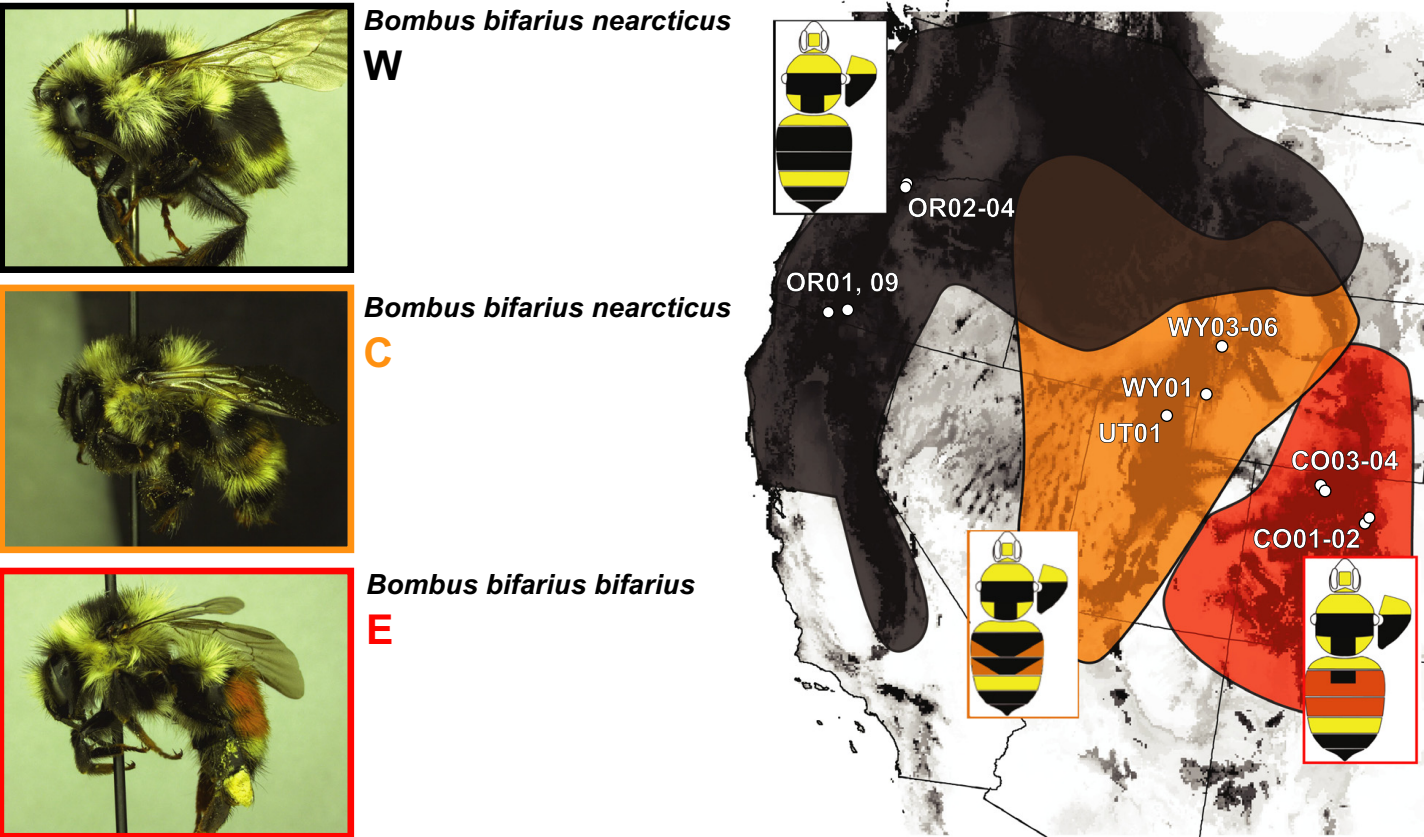
Geographic distribution of *Bombus bifarius* in the United States, with sampling sites and general approximation of regions where each color pattern is found (red: *B. bifarius bifarius‐*E red‐banded, black: *B. bifarius nearcticus*‐W black‐banded, orange: *B. bifarius nearcticus*‐C, reflecting regions of intermixed coloration). Boundaries are approximate, as individuals with intermixed pigmentation can be highly variable (Stephen 1957; Lozier et al. 2013; Williams et al. 2014). The underlying gray‐scale range prediction for *B. bifarius* comes from the ecological niche model of Lozier et al. (2013), where darker shades indicate higher predicted habitat suitability.

*Bombus* (*Pyrobombus*) *bifarius* Cresson is among the more dramatic cases of divergence in abdominal coloration in North American bumble bees (Stephen 1957; Williams et al. 2014) (Fig. 1). In the USA, two major color forms belong to two of the main North American regional color pattern groups (Plowright and Owen 1980; Williams 2007); populations in the easternmost parts of the species range in the southern Colorado Rocky Mountains, primarily in Colorado (CO) and southeastern Wyoming (WY), exhibit bright red hair color on the second and third abdominal tergites (red‐banded), while those in the westernmost Pacific region have black colored hairs (black‐banded). Geographically intermediate populations have much more variable pigmentation, usually with some degree of mixed coloration between the more extreme red‐ and black‐banded forms (Lozier et al. 2013; Williams et al. 2014) (Fig. 1). Subspecific names have been applied to reflect geographic color‐pattern differences (Stephen 1957): *B. bifarius bifarius* Cresson corresponds to the red‐banded form occurring primarily in easternmost populations (henceforth *bifarius‐*E), whereas *B. bifarius nearcticus* Handlirsch is more common and widespread, ranging from the black‐banded forms of the western‐most US (*nearcticus‐*W) but also including more intermediate mixed‐color forms from centrally located populations (*nearcticus‐*C) (Stephen 1957).

The evolutionary status of *B. bifarius* lineages remains unclear (Lozier et al. 2013; Williams et al. 2014). Microsatellites show structuring consistent with both phenotype and subspecies designations, distinguishing the eastern red‐banded *bifarius‐*E populations from the remaining *nearcticus* populations that show more complex patterns of genetic and phenotypic variation but generally cluster together (Lozier et al. 2011, 2013). However, fairly weak differentiation, mixed assignment of individuals to multiple genetic groups, and gradients in both allele frequency and pigmentation through intermediate populations all suggest the possibility of ongoing gene flow between two major population groups (Lozier et al. 2011, 2013). The relatively small numbers of markers analyzed may have limited the resolution of analyses, however, and the apparent connectivity could stem from weak drift in large regional bumble bee populations, combined with the high variability of microsatellites. Resolving relationships among color forms will be a key for revealing the evolutionary changes that contribute to phenotype in this species.

In this study, we clarify the relationships among lineage and color‐pattern differentiation across the major red‐vs‐black *B. bifarius* pigmentation groups of the contiguous USA, employing two complementary genomic approaches. Given the diversity of next generation sequencing methods available for evolutionary analysis, our goal was to strengthen conclusions by incorporating multiple sequencing and processing strategies, while also taking advantage of the unique information that might be provided by different genomic data sources. We perform transcriptome sequencing (RNAseq) (Renaut et al. 2010; Geraldes et al. 2011; De Wit et al. 2012; De Wit and Palumbi 2013) and double digest restriction enzyme associated DNA (ddRAD) sequencing (Davey et al. 2011; Peterson et al. 2012) to generate markers. ddRAD offers large numbers of short sequences adjacent to restriction cut sites throughout the genome and should reflect a relatively random sample of genomic variation. RNAseq generates large numbers of SNPs in expressed gene regions that may reveal distinct evolutionary patterns in protein coding regions compared to ddRAD sequences. We sequence representative samples of phenotypically distinguishable bees from CO (*bifarius‐*E) through Utah (UT) and WY (*nearcticus‐*C), and Oregon (OR) (*nearcticus‐*W) to investigate phylogeographic relationships among extreme and intermediate color forms and evaluate whether existing subspecies names accurately reflect distinct evolutionary lineages. We utilize this large number of loci to test whether divergence among color forms is limited or widespread across the genome, and to examine admixture hinted at by previous studies to determine whether phenotypically intermediate *nearcticus‐*C bees exhibit any hybrid ancestry between the more discrete *bifarius*‐E and *nearcticus‐*W phenotypes.

## Methods

### Samples

Microsatellite studies (Lozier et al. 2011, 2013) provide basic knowledge of range‐wide population structure of *B. bifarius* in the western USA. We shift the focus here to increased genome‐scale sampling to explore relationships among select representatives from the two extreme black‐ and red‐banded *B. bifarius* color forms and geographically and phenotypically intermediate bees (Fig. 1). Flying worker bees were sampled by sweep netting across a color pattern and spatial transect from Oregon, Wyoming, Utah, and Colorado (Fig. 1D; Table 1), and pooled into western (OR), central (WY/UT), and eastern (CO) populations based on geography and phenotype. With the focus of this study on expanded genomic coverage for coarse‐scale comparison of divergence and admixture across major lineages, rather than a fine‐scale analysis of population structure, samples should be representative of regional lineages for such “subspecies‐level” questions (Lozier et al. 2011, 2013). Samples were collected into RNAlater (Invitrogen, Carlsbad, CA), stored at 4°C overnight, transferred to dry ice, and stored at −80°C. RNA was isolated with RNeasy kits (Qiagen, Valencia, CA) or trizol (Invitrogen) from head and thorax tissues. DNA isolations for ddRAD sequencing (Peterson et al. 2012) were performed following a modified DNeasy protocol (Lozier 2014).

**Table 1:** Collection site information, including the population and phenotypic groupings used for population genetic analyses, and sample sizes (*N*) for RNA sequencing (RNAseq) and double digest restriction site associated DNA tag (ddRAD) sequencing used in analyses, after removal of putative siblings (see Methods)

Site	State	County	Latitude	Longitude	*B. bifarius* subsp. (T2‐3 color)^a^	*N* RNAseq	*N* ddRAD
CO01	Colorado	Boulder	39.994	−105.418	*bifarius*‐E (red)	1	1
CO02	Colorado	Boulder	39.940	−105.555	*bifarius*‐E (red)	2	8
CO03	Colorado	Routt	40.349	−106.750	*bifarius*‐E (red)	2	1
CO04	Colorado	Routt	40.448	−106.800	*bifarius*‐E (red)	1	1
OR01	Oregon	Klamath	42.396	−122.201	*nearcticus*‐W (black)	2	8
OR02	Oregon	Clackamas	45.301	−121.769	*nearcticus*‐W (black)	1	1
OR03	Oregon	Clackamas	45.331	−121.708	*nearcticus*‐W (black)	1	1
OR04	Oregon	Clackamas	45.256	−121.712	*nearcticus*‐W (black)	2	5
OR09	Oregon	Jackson	42.073	−122.754	*nearcticus*‐W (black)	1	1
UT01	Utah	Cache	41.735	−111.823	*nearcticus*‐C (mixed)	1	1
WY01	Wyoming	Lincoln	42.559	−110.895	*nearcticus*‐C (mixed)	–	1
WY03	Wyoming	Teton	43.657	−110.797	*nearcticus*‐C (mixed)	3	7
WY04	Wyoming	Teton	43.657	−110.790	*nearcticus*‐C (mixed)	3	4
WY06	Wyoming	Teton	43.669	−110.821	*nearcticus*‐C (mixed)	1	1

a T2‐3 coloration = primary pigment classification for the second and third abdominal tergites

Bumble bees are social and sampling sibs can produce artificial population structure, although collecting sister workers is relatively rare (Cameron et al. 2011) and we attempted to maximize independence of samples by collecting small numbers of bees from multiple localities to reflect each region (Table 1). Thus after preliminary SNP calling using all sequenced individuals (using methods below), we estimated pairwise relatedness within *bifarius‐*E, *nearcticus‐*W, and *nearcticus‐*C using VCFtools (Yang et al. 2010; Danecek et al. 2011). Relatedness estimates were ~1.0 for self and ~0.0 for the majority of non‐self pairs. A small number of non‐self pairs exhibited relatedness near or greater than 0.5, and as they were always from the same collection area, they were considered putative sisters, and we retained only one sample from each pair (RNAseq: only one bee removed from *nearcticus*‐C; RADseq: 1 removed from *bifarius‐*E, 2 from *nearcticus‐*W, 2 from *nearcticus‐*C). Ultimately, a total of 22 (including a single *B. vosnesenskii* worker as an out‐group) and 41 unrelated diploid workers were included in RNAseq and ddRAD experiments, respectively (Table 1), numbers that should prove sufficient for determining major lineage relationships with the large number of markers afforded by genome‐scale data (Felsenstein 2006; Novembre & Ramachandran 2011; Rheindt et al. 2014).

### RNAseq analysis

Transcriptome libraries were generated by the Hudson Alpha Institute for Biotechnology (Huntsville, AL) from 500 ng total RNA, and run on an Illumina HiSeq 2000 with 100 bp paired‐end chemistry, producing 17.2 × 10^6^ (1.9 × 10^6^ S.D.) reads per individual (Appendix S1). Trim Galore! (http://www.bioinformatics.babraham.ac.uk/projects/trim_galore/) was used for quality control and stringent removal of adapter sequence, low‐quality bases (<20), and short reads (<20 bp). Samples were aligned to the *B. impatiens* 2.0 genome scaffolds available on RefSeq (bim_ref_BIMP_2.0_chrUn.fa; Sadd et al. 2015). *B. impatiens* is a close relative of *B. bifarius* and *B. vosnesenskii* (Cameron et al. 2007) and the high synteny of *Bombus* genomes across deeper phylogenetic timescales than considered here (Sadd et al. 2015) should facilitate accurate alignment and the use of *B. impatiens* as an out‐group in certain analyses. TopHat v2.0.10 (Kim et al. 2013) was used for alignments. We experimented with allowed mismatches to evaluate the balance between misalignments (too relaxed) and missed alignments (too stringent). Read mapping differed only by a few percent between different mismatch values, and preliminary analyses with different parameters produced similar results and conclusions to those presented here, and we ultimately selected three mismatches per read. Some stringency was considered preferable to allowing too many false alignments, while still producing good mapping percentages (Appendix S1) and sufficient SNPs. Following recommendations in Quinn et al. (2013), we excluded reads with map quality <20 in SAMtools v0.1.19 (Li et al. 2009) and marked and removed duplicate reads with Picard (http://broadinstitute.github.io/picard).

#### RNAseq variant detection

SNPs were called once for both *B. bifarius* and *B. vosnesenskii*, and then separately with *B. vosnesenskii* excluded. Samtools (mpileup, bcftools, and varFilter; Li et al. 2009) was used for variant calling (Quinn et al. 2013; Singhal 2013), ignoring indels and SNPs within 4 bp of a gap. To further minimize artifacts associated with RNAseq SNP calling, we used vcftools to filter variants. The final data sets comprised 91,282 SNPs that were variable across the three species and 46,259 SNPs that were variable within *B. bifarius* alone, each with ≥12× coverage per sample (more stringent, e.g., 20×, filters did not substantially alter mean SNP depth or final results), tested for excess heterozygosity (e.g., Taylor et al. 2014) in either *bifarius‐*E or *nearcticus‐*W (*nearcticus‐*C populations were not screened to allow for the possibility of admixture generating many heterozygotes), and with ≤20% missing data. Although linkage is weak in bees (Whitfield et al. 2006; Stolle et al. 2011; Sadd et al. 2015), for certain analyses where unlinked SNPs would be beneficial, the data set was thinned to one variant per 20 kb. For some analyses, SNPs were classified as synonymous or nonsynonymous from *B. impatiens* 2.0 genome annotations using SNPeff (Cingolani et al. 2012). With the caveat that the *B. impatiens* draft is not perfectly annotated and annotations may not always transfer across species, SNPs falling outside of any *B. impatiens* exons or that produced SNPeff warnings (e.g., from incomplete or incorrect annotations) were excluded from classification; visual inspections of alignments suggested that remaining sites appeared accurately classified.

#### Analysis of RNAseq variants

Data manipulation for some analyses relied on scripts from De Wit et al. (2012) and PGDSpider (Lischer and Excoffier 2012). To identify signatures of average differentiation and admixture at the individual level, we performed principal components analysis (PCA) on RNAseq SNPs using smartpca and twstats (Patterson et al. 2006; Price et al. 2006) after removing all missing data. To quantify average differentiation at the population level, pairwise population *F*_ST_ [SNPs with minor allele frequency (MAF) > 0.05] was calculated with Arlequin 3.5 (Excoffier and Lischer 2010), with significance tested by 10,000 permutations. To distinguish whether the patterns of differentiation or admixture were restricted or widespread across the genome, SNP‐specific *F*_ST_ values, sliding window analysis, and allele frequencies were calculated in vcftools. As a final test to determine whether the intermediate phenotype of *nearcticus*‐C could be explained by admixture between the more discrete color forms, three‐population tests of admixture in *nearcticus*‐C were conducted in Treemix 1.2 (Pickrell and Pritchard 2012) for the same data sets using the *f*_3_ statistic (Reich et al. 2009; Patterson et al. 2012) with the form *f*_3_(*nearcticus‐*C; *bifarius‐*E, *nearcticus*‐W).

To better describe phylogeographic relationships and quantify relative divergence among lineages, we performed two phylogenetic analyses on the RNAseq SNPs. First, SNPs were concatenated for maximum likelihood analysis with RAxML 8.0.20 (Stamatakis 2014) with 100 bootstrap replicates. To take advantage of SNP‐only models in RAxML, sites were converted to FASTA format, heterozygous sites coded as missing data, and invariant sites removed (Final *N *=* *52,081 SNPs) as described in the manual. As suggested for SNP‐only data in RAxML, we used the general time reversible model, a gamma model of rate heterogeneity and ascertainment correction, and *B. vosnesenskii* and *B. impatiens* as out‐groups. The final tree was largely unaffected by the exact method of sample processing (e.g., removing heterozygous sites, including all SNPs vs. thinned data) or the model employed. Forcing tree‐like structures is not always ideal for individuals, and concatenation violates the reality that independent gene trees underlie unlinked loci, but phylogenetic analysis can provide an overall distance among individuals and populations with respect to *B. impatiens* and *B. vosnesenskii* (e.g., Rheindt et al. 2014; Soria‐Carrasco et al. 2014). Such problems can also be alleviated by modeling population‐level relationships and evolutionary parameters within a coalescent framework (Bryant et al. 2012; Fujita et al. 2012). Thus, we used the Bayesian coalescent species tree method SNAPP (Bryant et al. 2012), which is designed to estimate relationships from unlinked bi‐allelic data, to gauge relative divergence times. We utilized only synonymous sites (likely to be closest to neutral) with no missing data from the unlinked 20 kb‐thinned SNP set. This considerably improved the performance of SNAPP without sacrificing the inferred topology, which, as for RAxML, was stable in a variety of test data sets with greater or fewer numbers of loci (not shown). SNPs were coded as “0 = homozygous derived allele”, “1 = heterozygote”, and “2 = homozygous *B. impatiens* allele.” We estimated the *u* and *v* parameters from the data (Drummond and Bouckaert 2014). Other parameters were set as default, except for exploring sensitivity to the gamma distribution priors on *θ = 3N*_e_ *μ* (for haplodiploids) by adjusting the *α* and *β* parameters to account for a range of possible population sizes (e.g., Rheindt et al. 2014). Because of demographic parameter conversion uncertainty, only relative divergences are presented, but topologies were unaffected by parameter settings. For default priors, 5 × 10^5^ generations (with 10% burn‐in) was sufficient to produce effective sample sizes of 200 in Tracer 1.6 (Rambaut and Drummond 2013); however, alterations to default priors required increases to >1.5 × 10^6^ generations in some runs. Results were qualitatively similar regardless of run length.

As a final test of the relative contributions from divergence and admixture to the evolution of *B. bifarius* lineages, we made use of the inferred phylogenetic relationships for demographic modeling with *∂a∂i* v1.7 (Gutenkunst et al. 2009) under a three‐population isolation with migration model allowing population size changes and symmetrical migration. The folded site frequency spectra were estimated with the full *B. bifarius*‐only SNP set projected down to 12 sampled chromosomes per population. No parameter rescaling was performed, as we were only interested in comparisons of relative migration rates between the three populations.

### Double digest RADtag (ddRAD) sequencing

#### ddRAD libraries and sequencing

ddRAD sequencing allowed increased sampling for each lineage and provides a complementary source of genomic data to RNAseq that should more randomly sample the genome. We prepared libraries following Peterson et al. (2012). Genomic DNA (250 ng) was digested with 1 *μ*L each *Eco*RI (100,000 U/*μ*L) and *Msp*I (20,000 U/*μ*L microliter) (New England Biolabs, Ipswitch, MA) following manufacturer protocol. Digested samples were cleaned with Agencourt Ampure XP beads (Beckman Coulter, Danvers, MA). Ligation of double stranded adapters with unique inline barcodes followed Peterson et al. (2012). Barcoded samples were pooled and 225–375 bp regions were selected using a Pippin Prep (Sage Science) 2% agarose gel cassette and confirmed via PCR and electrophoresis. Size‐selected fragments were purified with M‐270 Streptavidin Dynabeads (Invitrogen). To incorporate primer regions for Illumina sequencing libraries, the size‐selected product was split into five replicates and amplified with Phusion High‐Fidelity DNA polymerase (manufacturer's protocol) for 12 cycles. Products were pooled and Ampure purified. The library was sequenced on the Illumina MiSeq platform (150 bp chemistry) at Hudson Alpha.

#### ddRAD sequence processing

Sequences were demultiplexed and cleaned using Stacks v1.23 (Catchen et al. 2013) *process_radtags*. Reads with multiple errors in the barcode and cut site and with average Phred quality score <10 within a sliding window (default 15% of total read length) were discarded. Adapter sequences were identified and removed and all reads trimmed to 135 bp (shorter reads excluded). We elected to construct ddRAD loci de novo and treat them as random genomic marker data, as is common in diverse genome reduction genotyping studies (e.g., Emerson et al. 2010; Baxter et al. 2011; Gompert et al. 2012; Catchen et al. 2013; Benestan et al. 2015), and to provide a contrasting approach for comparison to the *B. impatiens*‐aligned RNAseq data. Loci were created with the Stacks *denovo_map* pipeline using only the *Eco*RI end fragment. Removing the MspI read excludes some data, however, using only a single end produced sufficient markers for our goals, simplified bioinformatics in the presence of both overlapping and non‐overlapping read pairs, and reduced the numbers of closely‐spaced SNPs that should provide minimal novel information. Stacks implements approaches to filter erroneous genotypes during de novo alignment (Catchen et al. 2013), and although we did not use *B. impatiens* for alignments, we did employ the genome post hoc to optimize parameters for excluding artifacts such as over‐merged (e.g., potential repeat elements and paralogs) and under‐merged (e.g., highly divergent alleles) loci. Following an approach similar to (Catchen et al. 2013), we tested different parameters for mismatches allowed between stacks within and among individuals to optimize merging of loci, and ran 10 batches ranging from 1 to 10 allowed mismatches. For each parameter set, we used Stacks *populations* to identify loci present in >50% of the samples, and BLASTN (E‐value of 1e‐10) to align against the *B. impatiens* 2.0 genome (Sadd et al. 2015) in Geneious v7 (Biomatters; http://www.geneious.com). We removed sequences that matched >1 region over >75 bp in the reference genome (putative overmerged loci) and where multiple loci matched a single reference position (undermerged loci). Although we did not use genome‐aligned data for SNP calling and thus do not make use of position data, this genome‐assisted approach should ensure a set of unique allelic markers. Four mismatches allowed per 135 bp produced the most unique ddRAD loci and was used for the final analysis, although numbers of loci were similar across a range of Stacks parameter settings. The final locus set containing uniquely matching loci (91.8% of initial ddRAD loci) was specified as a white list for a final Stacks *populations* run to generate output files for 41 individuals at SNPs with ≥15× depth of coverage (*N* = 37,848 SNPs). We used vcftools to filter the data set containing all SNPs with <20% missing data (*N *=* *25,308 SNPs).

Calculation of *F*_ST_, *f*_3_, and allele frequencies used Arlequin, Treemix, and vcftools, as above. To test for admixture, major groups of populations were identified using STRUCTURE (Falush et al. 2003) with a data set filtered to contain only one SNP per ddRAD locus to minimize within‐locus linkage (7248 SNPs). We used the admixture model with 100,000 iterations (burn‐in of 25,000 iterations). We allowed the number of populations (*K*) to range from 2 to 5 and performed 2 replicate iterations each. Results were processed with STRUCTURE HARVESTER (Earl and VonHoldt 2012) to determine the optimal value of *K* with Δ*K* (Evanno et al. 2005). We used SPLITSTREE v4.13.1 (Huson and Bryant 2006) to construct a phylogenetic network, implementing the neighbor‐net (ordinary least squares variance) and equal angle algorithms, using uncorrected *p‐*distances with heterozygous ambiguities averaged and normalized, and 1,000 bootstrap replicates (>75% shown). Analysis with *∂a∂i* was performed as above with a projection to 16 alleles per population.

## Results

### Sequencing summary

Approximately 2 × 10^8^ 100 bp RNAseq read pairs were included for samples in our analysis, with an average of 97% passing quality control and, of these, an average of 76% of reads per sample aligned to the *B. impatiens* genome for final analysis (Appendix S1). RNAseq‐derived SNPs that passed quality filters were sequenced to an average of 66× coverage per site per sample (Appendix S2), with samples receiving fairly even coverage ranging from an average of 48×–78× per SNP. Approximately 3 × 10^7^ ddRAD reads were obtained (Appendix S3). After processing and removal of putative siblings and quality control filtering, an average of 91% of these 135 bp reads were retained, with a mean of 31× after removing loci with >20% missing data (*N *=* *25,308; Appendix S2).

### Phylogenetic and ancestry clustering analyses

**Figure 2:**
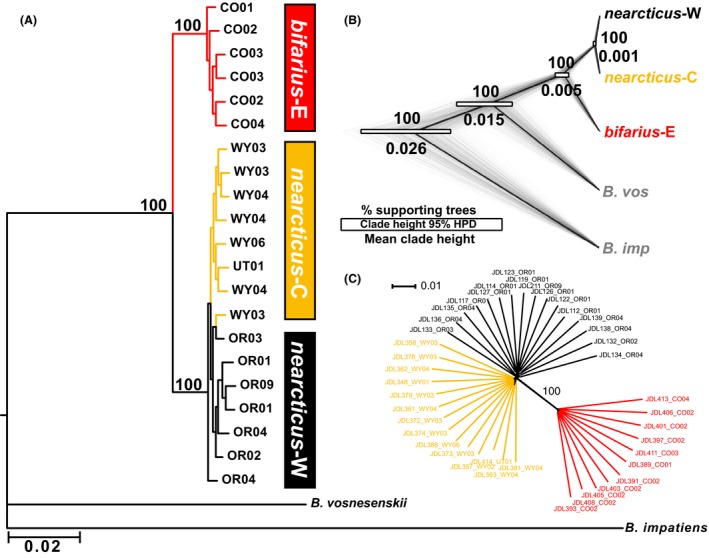
(A) RAxML best‐scoring likelihood tree for *B. bifarius*, with *B. vosensenskii* and the *B. impatiens* genome sequence as out‐groups, from 52,081 SNPs, with bootstrap support for major clades. (B) Consensus coalescent species tree (dark line) and 450 individual replicate trees (gray lines) from 1568 synonymous RNAseq SNPs (thinned every 20 kb) for default prior parameter settings, with node support and mean scaled divergence time with 95% highest posterior density, drawn with Densitree (Bouckaert 2010). (C) Split network (uncorrected *P‐*distances, ambiguous states averaged) constructed from concatenated ddRAD SNPs.

Phylogenetic analysis of concatenated RNAseq *B. bifarius* SNP data (Fig. 2A) produces two major groups: one monophyletic group comprising red‐banded *bifarius*‐E bees, and the second comprising *nearcticus* (100% support). Apart from a single sample, *nearcticus*‐W and *nearcticus‐*C form their own groups, however, this split is not well supported. SNAPP produces a similar population tree (Fig. 2B), with *bifarius*‐E sister to the more closely related *nearcticus*‐W and *nearcticus*‐C, with all nodes supported with a posterior probability of 1.00. Absolute divergence and *θ* estimates were sensitive to *θ* prior parameters (*α* and *β*), but, as observed in other studies (Rheindt et al. 2014), topology and support were identical across test runs and divergence time ratios for clades were relatively similar (e.g., Appendix S4). The greatest uncertainty surrounds divergences of *B. impatiens* and *B. vosnesenskii*, as expected given the more limited population sampling of these lineages. However, the *B. bifarius* population tree and divergence time ratio is similar with out‐group removal (not shown). Notably, the divergence time confidence region for *bifarius*‐W and the two *nearcticus* lineages does not overlap that within *nearcticus*. Split network analysis of ddRAD SNPs also produces two major clades (Fig. 2C), one containing only *bifarius*‐E and one containing members of *nearcticus*‐W and *nearcticus*‐C with substantial distance between the two clades (RNAseq produced similar results; Appendix S5).

**Figure 3:**
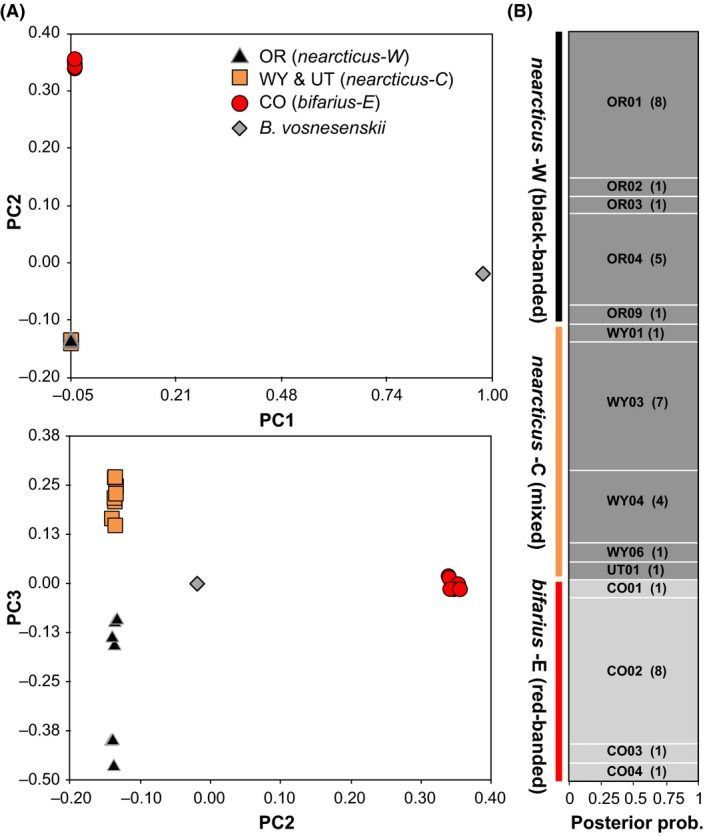
(A) Principal components axes 1 and 2 (upper) and 2 and 3 (lower) using the full RNA SNP data set filtered for missing data (*N* = 45,089 SNPs) for *B. bifarius* and *B. vosnesenskii*. (B) STRUCTURE results for ddRAD seq data, for *K *=* *2 one SNP per ddRAD locus, no minor allele frequency filter, polymorphic loci only (*N* = 7248 SNPs). Horizontal bars show posterior assignment probabilities of each individual (vertical axis) to a particular cluster, based on the proportion of the vertical bar that contains each color. Population of origin indicated and collection sites with number of specimens collected in parentheses.

Clustering analyses also divide *B. bifarius* into two major groups, with no clear evidence of mixed ancestry between *bifarius* and *nearcticus* populations (Fig. 3). The first axis (eigenvalue = 12.6) in the RNAseq PCA (Fig. 3A) separates *B. vosnesenskii* from *B. bifarius* and the second (eigenvalue = 2.6) separates *bifarius*‐E and *nearcticus* W + C (*P *<* *0.001). The third axis (eigenvalue = 0.4) separates *nearcticus‐*W from *nearcticus‐*C (*P *<* *0.001), with some weak subdivisions among *nearcticus*‐W into northern and southern OR clusters also observed, a pattern that may suggest within‐subspecies isolation by distance (IBD). A similar pattern appears in a PCA using the 20 kb distance‐filtered SNP set, but with weaker within‐*nearcticus* resolution (Appendix S6). The ddRAD STRUCTURE analysis (Fig. 3B) identifies two clusters (Fig. 3B, Appendix S7), with *nearcticus*‐W + *nearcticus*‐C populations in one cluster, and *bifarius*‐E in another, with no admixed genotypes. Setting *K *=* *3 produces no additional structuring. A second STRUCTURE analysis was performed only for *nearcticus* samples, but no additional subdivision was detected (Appendix S7). STRUCTURE analysis for RNAseq SNPs produced essentially the same *K *=* *2 pattern as the ddRAD data, failing to show any within‐*nearcticus* separation (not shown). Demographic modeling with *∂a∂i* generally supports the distinction between *bifarius* and *nearcticus* groups, with near‐zero estimates of contemporary migration relative to the high levels between *nearcticus* populations (only ~1% of that estimated between the two *nearcticus* populations) (Appendix S8).

### Population genetics

**Figure 4:**
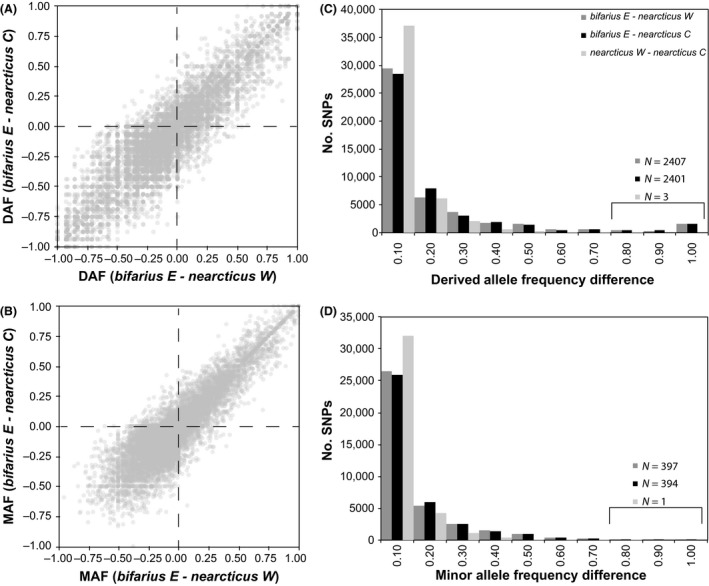
Allele frequency comparison for the three lineages. Correlation scatterplots for (A) DAF differences between *bifarius*‐E and each *nearcticus* lineage at each of 46,259 RNAseq SNPs and (B) global MAF differences between *bifarius*‐E and each *nearcticus* lineage at each of 37,848 ddRAD SNPs. Histograms of allele frequency differences for all three‐population pairs for (C) RNAseq SNP DAFs and (D) ddRAD SNP MAFs.

Population genetic analyses support the split between *bifarius* and *nearcticus* bees revealed by the tree‐based and clustering analyses. Red‐banded *bifarius*‐E are equally divergent from intermediate *nearcticus‐*C and black‐banded *nearcticus‐*W for both RNAseq and ddRAD SNPs (Fig. 4). For the RNAseq SNPs, derived (i.e., non‐*B. impatiens*) allele frequency (DAF) differences between *bifarius*‐E and both *nearcticus* populations are strongly correlated (Fig. 4A; Pearson's *r *=* *0.93). Both pairs exhibit an excess of SNPs with fixed allele frequency differences compared to those observed for *nearcticus‐*W versus *nearcticus‐*C (Fig. 4C), which show a relative deficit of large DAF differences and a much higher frequency of SNPs with little DAF difference. There was no pattern with respect to fixation of ancestral (i.e., *B. impatiens* reference) or derived alleles between *nearcticus* and *bifarius*‐E, with similar numbers of SNPs in both categories (e.g., 709 fixed ancestral and 753 fixed derived alleles in the *bifarius*‐E versus *nearcticus*‐W comparison). Similar correlations for differences in global MAF are apparent for the ddRAD SNPs (Fig. 4B; Pearson's *r *=* *0.89). A lower frequency of fixed‐divergence SNPs between *bifarius‐*E and the *nearcticus* populations is observed in the ddRAD than in the RNAseq data; however, there is still a substantial excess of SNPs with high‐frequency differences and a deficit of SNPs with low‐frequency differences, relative to that between the two *nearcticus* populations (Fig. 4D). Consequently, in both RNAseq and ddRAD data average, *F*_ST_ is low between the *nearcticus* populations and high in *bifarius*‐E versus *nearcticus* pairs (Table 2). The *nearcticus*‐W versus *nearcticus*‐C *F*_ST_ values are comparably low for both SNP sets; however, *F*_ST_ values for *bifarius*‐E versus *nearcticus* comparisons are notably higher for the RNAseq SNPs, particularly for nonsynonymous RNAseq SNPs. A randomly thinned SNP set also exhibits somewhat elevated *F*_ST_'s, however, suggesting this increase may be a random effect from reducing the number of SNPs analyzed. Notably, regardless of filtering or data set, very little variation in *F*_ST_ for the two *nearcticus* populations is observed, with most variability apparent in the *bifarius*–*nearcticus* comparisons.

**Table 2:** Pairwise *F*_ST_ and the three‐population *f*_3_ statistic testing for mixture in *nearcticus*‐C for different SNP sets derived from RNA sequencing (RNAseq) and double digest restriction site associated DNA tag (ddRAD) sequencing

	No. SNPs^1^	*nearcticus‐W* versus *nearcticus‐C*	*nearcticus‐W* versus *bifarius‐E*	*nearcticus‐C* versus *bifarius‐E*	*f*_3_ (*nearcticus‐C; bifarius‐E, nearcticus‐W*)^2^
RNAseq‐all SNPs					
All Sites	20,170	0.015***	0.449***	0.445***	0.002
Synonymous Sites	7450	0.013***	0.444***	0.440***	0.002
Non‐synonymous Sites	2016	0.021***	0.597***	0.592***	0.003
RNAseq‐20 kb thinned					
All Sites	2251	0.018***	0.538***	0.529***	0.002
ddRAD‐SNPs					
All SNPs	7948	0.019***	0.243***	0.238***	0.001
Single SNP per locus	2115	0.019***	0.258***	0.252***	0.001

^1^Single‐nucleotide polymorphism with >0.05 minor allele frequencies were used in *F*_ST_ calculations. Otherwise data sets are as described in Methods.

^2^A negative *f*_3_ would indicate admixture in *nearcticus*‐C from both *nearcticus*‐W and *bifarius*‐E, while a positive value is expected under no mixture; statistical tests of *f*_3_ are only informative for negative values, but in no instance supported negative *f*_3_ values.

***Significantly greater than zero, *P *<* *0.001.

The *f*_3_‐statistics are positive in all data sets, providing no support for admixture in *nearcticus*‐C leading to deviations from a three‐population tree (Table 2). To further explore for signals of admixture, however, we examined *F*_ST_ at SNPs that were diagnostic (*F*_ST_ = 1) for the hypothesized “parental” populations (*bifarius‐E* vs. *nearcticus*‐W) in the two remaining population pairs. For the 1341 diagnostic RNAseq SNPs, mean *F*_ST_ = 0.002 for *nearcticus*‐W versus *nearcticus*‐C and 0.991 for *bifarius*‐E versus *nearcticus*‐C (and 0.003 and 0.992, respectively, for diagnostic ddRAD SNPs). Thus, SNPs that are fixed between the two extreme color forms are also at or near fixation between *bifarius*‐E and *nearcticus*‐C. Taking advantage of the *B. impatiens* genome alignment, we examined whether RNAseq SNPs with high frequency differentiation were restricted within the genome with sliding window analysis across scaffolds (non‐overlapping window size = 20 kb). Regions of high differentiation (windows with *F*_ST_ ≥ 0.9) are scattered throughout the *B. impatiens* scaffolds; 196 windows across 101 scaffolds show high differentiation for *bifarius‐*E versus *nearcticus‐*W, 186 windows across 98 scaffolds show high differentiation for *bifarius‐*E versus *nearcticus‐*C, and no windows exhibit high divergence for the two *nearcticus* populations (Appendix S9).

## Discussion

The origins of insect color polymorphism is a topic of immense interest, especially the mechanisms by which intraspecific population diverge, while interspecific populations converge, on particular phenotypes, and the role such patterns have on diversification (Mallet and Joron 1999; Williams 2007; Jiggins 2008; Hines et al. 2011; Kronforst and Papa 2015). As Stephen (1957) noted regarding *B. bifarius*, “this highly variable species has undoubtedly caused more consternation to bumble bee taxonomists than any other species in western North America.” Morphological analyses of *B. bifarius* have been confounded by intermediate phenotypes that lie between the typical extreme red‐banded (*B. b. bifarius*) and black‐banded (*B. b. nearcticus*) color forms (Stephen 1957; Lozier et al. 2013; Williams et al. 2014) in the western USA. Although challenging for taxonomists, the relationships among both discrete and more intermediate color forms have the potential to be of great utility for understanding the strength of and mechanisms underlying genetic isolation and the correlations with phenotypic variation.

Previous microsatellite‐based studies indicated a moderate level of structure for two major *B. bifarius* groups, but with most individuals, especially geographic and phenotypic intermediates, exhibiting detectable contributions from both lineages that suggested ongoing gene flow (Lozier et al. 2011, 2013). However, results here provide no clear evidence for extensive genomic introgression as a major explanation for intermediate phenotypes. Instead, *B. b. bifarius* (red‐banded *bifarius‐*E) appear as a clearly divergent lineage from the more phenotypically variable *B. b. nearcticus* from OR (black‐banded *nearcticus*‐W) and from WY or UT (mixed colored *nearcticus*‐C), while the latter show a high genome‐wide similarity irrespective of color‐pattern variability. Across classes of SNPs, allele frequencies were similar within *nearcticus*, despite the geographic distance between sites, and high in both *nearcticus*‐*bifarius* comparisons, a pattern inconsistent with IBD or other types of pervasive admixture between the phenotypic extremes in more central localities (Fig. 3; Table 2). The *f*_3_ statistics are consistent with a three‐population tree without admixture, and individual‐based analyses from the SNP sets support *F*_ST_ patterns (Fig. 3), grouping *nearcticus‐*W + *nearcticus*‐C separate from *bifarius*‐E, with no evidence of hybrid genotypes.

Phylogenetic analysis likewise groups genotypes into two major clades consistent with existing subspecies classification, with weaker within‐*nearcticus* division, and provides some perspectives on the seemingly deep nucleotide divergence between the main *B. bifarius* lineages. We did not conduct a formal dating analysis at this point given challenges in estimating heterogeneous evolutionary rates across classes of SNPs. However, a divergence from *B. impatiens* approximately ~6.2 mya from calibrated phylogenetic analysis (Hines 2008a,b) applied to the coalescent tree here would suggest a rough mean divergence of the major *B. bifarius* lineages of ~0.96–1.2 mya (converted from range of means using different SNAPP priors; Appendix S4). Because of uncertainty in mutation rate variation and parameter settings, model simplicity, and limited out‐group sampling, this approximation should be viewed with caution. However, combined with other results that provide little evidence for admixture, including PCA, STRUCTURE, and the large percentage of fixed‐divergence SNPs, a mid‐late Pleistocene *B. bifarius* split is likely to be reasonable, even if the exact date is uncertain. The basic patterns of subspecific divergence and relationships developed here lay the groundwork for future studies to test specific phylogeographic hypotheses and refine divergence time estimates.

### Possible signatures of adaptive divergence or introgression?

Highly divergent regions in both *bifarius* vs. *nearcticus* comparisons were distributed across genomic scaffolds, suggesting extensive divergence across the genome rather than localized differentiation that might indicate islands of recent adaptive differentiation. These SNPs may represent a mixture of genome regions targeted by divergent selection, but to a large extent likely reflect neutral differentiation from population history and random drift (Nosil et al. 2009, 2012), which will make identifying the signatures of selection through standard “outlier” divergence mapping a challenge. However, while the broadly comparable patterns of structure for RNAseq SNP classes and ddRAD sites suggests general inferences about differentiation and admixture are robust, the increased differentiation at non‐synonymous SNPs could point to the action of divergent selection in genic regions. Future research will examine possible explanations for the differences in the degree of differentiation among different SNP classes, in particular focusing on regions containing many high‐divergence SNPs that may reveal signatures of positive selection.

Given these patterns of parallel genome‐wide divergence between *bifarius*‐E and both *nearcticus‐*W and *nearcticus‐*C, however, additional research will be needed to understand the balance between selection and reproductive isolation in maintaining the pigmentation dimorphism in *B. bifarius*. Previous results suggest that hot and dry low elevation landscapes act to drive differentiation at a regional scale in *B. bifarius* (Lozier et al. 2013), and it is likely that differential selection on pigmentation during allopatric divergence could have driven initial adaptive differentiation in the two extreme color forms. However, it is possible that color patterns could have played a more direct role in reproductive isolation (Jiggins et al. 2001), and as with other mimicry systems multiple hypotheses are plausible (Brown et al. 1974; Mallet and Singer 1987; Mallet and Joron 1999). The lack of clear genetic divergence between *nearcticus* populations certainly raises questions as to why bees with partial red‐banded pigmentation dominate many parts of the *B. b. nearcticus* range (Fig. 1). In general, red‐black coloration in *Bombus* is regulated as part of the melanin biosynthesis pathway (Hines 2008a; Rapti et al. 2014). Certain red/black *Bombus* color polymorphisms, most notably in the western NAm species *B. melanopygus*, seem compatible with single‐locus Mendelian inheritance (Owen and Plowright 1980), but for species like *B. melanopygus*, binning individuals into classes is relatively straightforward, there is little population structure, and the morphs are at least partly sympatric (Owen et al. 2010). Color in *B. bifarius* may be more complex, involving both historical phylogeographic isolation and a range of phenotypic intermediates. A simple single‐locus system may thus not explain pigmentation in this case, and more complex regulation of expression within the melanin pathway may be at play (Hines 2008a), including regulatory mutations or environmental effects that affect the timing of melanization.

Previous data originally led us to hypothesize an admixture contribution for the mixed *nearcticus*‐C phenotype, which would have led to clear hypotheses to detect loci involved in determining red pigmentation. In this scenario, an admixture outlier mapping approach could have provided clues about regions involved in pigmentation through adaptive introgression between lineages (i.e., evidenced by rare loci with *bifarius‐*E alleles at high frequencies in *nearcticus‐*C) (Buerkle and Lexer 2008; Crawford and Nielsen 2013; Hedrick 2013; Pallares et al. 2014). Such patterns have been observed, for example, at mimetic loci in *Heliconius* (*Heliconius* Genome Consortium 2012; Pardo‐Diaz et al. [Link]; Martin et al. 2013). However, no obvious candidate regions with unusually large similarity between *nearcticus*‐C and *bifarius*‐E were identified; sites that were highly differentiated between the more discrete color morphs were equally divergent between *bifarius*‐E and *nearcticus*‐C. For both RNAseq and ddRAD data sets, some individual SNPs showed slightly greater similarity in allele frequencies between *bifarius*‐E and *nearcticus*‐C, but a similar number of loci show equally high similarity for *bifarius*‐E and *nearcticus*‐W (Fig. 4), a pattern expected under divergence and genetic drift. Such patterns will make it challenging to distinguish any potentially adaptive introgression from background noise with population genetics alone.

In light of the unexpectedly large level of background divergence and lack of any clear admixture, we thus elected not to pursue genome “outlier” scans to avoid the risk of false positives owing to population structure. However, lack of evidence for obvious signatures of adaptation does not mean such loci do not exist. Transcriptomes of workers will lack genes expressed only in developing bees, and ddRAD data may be insufficiently dense for mapping large, outbred populations. In *Heliconius* mimicry complexes, only markers closely linked to localized genome changes that regulate red coloration exhibit evolutionary histories reflecting phenotype, with most genome regions reflecting demography (Hines et al. 2011). Denser genomic coverage may thus be needed for fine mapping color‐determining loci in *B. bifarius*. Together with such population genomic approaches, developing laboratory crosses may prove especially valuable for determining the degree to which the divergence observed here reflects pre‐mating barriers to reproduction, the phenotypes observed in true hybrids, and ultimately for linkage analyses of pigmentation genes.

### Differences between NGS and microsatellite conclusions

As population genomic studies accumulate, it will be interesting to observe how frequently studies exhibit subtle deviations from previous hypothesis such as those observed here. Instead of reflecting substantial admixture, microsatellites may have poor statistical power for resolving the strength of population structure in *B. bifarius*. The microsatellites used previously have high polymorphism (mean gene diversity = 0.77), limiting the degree of possible differentiation that can be detected by *F*_ST_ or analogous measures (e.g., Jost 2008; Meirmans and Hedrick 2011). Indeed, differentiation statistics that account for polymorphism (e.g., Jost's D; Jost 2008) were somewhat closer to levels reported here (Lozier et al. 2011). Further, the high diversity is indicative of large populations (or high mutation rates) that may be influenced by incomplete lineage sorting, homoplasy, or other factors that could limit differentiation, and could be overcome by the large number of SNPs examined here even with smaller sample sizes. Alternatively, signatures of hybridization may be complex (e.g., Gompert et al. 2014; Cahill et al. 2015; Lindtke and Buerkle 2015), and it is possible that our focus on extensive genomic sampling of representative exemplar populations could oversimplify the system. Strong genetic clustering can, for example, be observed with incomplete sampling in IBD populations (Meirmans 2012). The degrees of differentiation here argue against this (see above), and bees from widely separated sites within population groups cluster together as well as individuals from the same locality (Figs. 2 and 3). The samples thus appear representative of broader regional diversity, and the main conclusions are not likely driven by local population structure or uneven sampling, but instead by deeper phylogenetic structuring. Nonetheless, admixture is a complex process, and although finer‐scale “genomics‐quality” samples were not available for this study, increased spatial sampling may ultimately reveal localized hybridization. This study demonstrates, however, that admixture is not sufficiently extensive to leave clear signatures across the genome in all intermediate populations, even where mixed‐color phenotypes might suggest a history of possible hybridization. It will likely be necessary to combine both population genomic and spatially intensive sampling to ultimately resolve such discrepancies.

## Conclusions

This study highlights the novel insights that can come from expanding genomic data sets for resolving evolutionary relationships among closely related lineages and illustrates that different types of genetic data may lead to similar but subtly different conclusions. Genome‐wide patterns suggest a history of long isolation between major *B. bifarius* lineages, which strengthens previous data on the role of geographic and climatic barriers to dispersal as a driver of population structure, but suggests even less gene flow than previously hypothesized. Results thus hold significance for future taxonomic work, but also for understanding the processes involved in maintaining divergence in widespread species. More intensive morphological analysis, mating experiments, and analysis of other color variants, especially individuals with partially red coloration within the *B. b. nearcticus* range (Stephen 1957), should be conducted to ultimately determine the need for taxonomic revision, the relative rates at which species and phenotypes diverge, and the population and genome‐level mechanisms underlying reproductive isolation (e.g., Lindtke and Buerkle 2015). We are only beginning to understand the phylogeographic relationships within this remarkably complex species, and adaptive pigmentation in bumble bees generally. Together with laboratory crosses, denser genome sequencing, and functional genomics studies, the more refined understanding of relationships among *B. bifarius* lineages presented here will provide the framework for developing general hypotheses for the evolutionary processes underlying red‐black polymorphism in bumble bees that can be tested in other species.

## Data Accessibility

DRYAD submission (http://dx.doi.org/10.5061/dryad.k21r5) contains vcf formatted files containing SNP data for both ddRAD and RNAseq data, fasta files used for phylogenetic analyses, input XML for SNAPP (default priors), BAM‐formatted RNAseq alignments and ddRAD data files.

## Conflict of Interest

None declared.

## Supporting information


**Appendix S1.** RNA Illumina sequencing coverage for each sample used in analyses, including total numbers of reads (averaged of the two paired ends), percentage passing TrimGalore! quality control, and the percentage successfully mapped by Tophat with a mismatch setting = 3 per read.
**Appendix S2.** (A) Distribution of sequencing coverage per SNP per individual for the 46,259 *B. bifarius* RNAseq SNPs, with a mean = 66× (69 SD) coverage per SNP per individual. (B) Distribution of sequencing coverage per SNP per individual for the 25,308 *B. bifarius* ddRAD SNPs, with a mean = 31× (9 SD) coverage per SNP per individual.
**Appendix S3.** Illumina sequencing information for ddRAD samples used in analysis, with total number of reads and number and percentage of reads retained after quality control (QC) with Stacks *process_radtags*.
**Appendix S4.** Examples of sensitivity of node ages for SNAPP clades explored under different *α* and *β* prior settings, showing changes in absolute node age, but stability of relative node ages.
**Appendix S5.** Phlyogenetic network for 46,259 RNAseq SNPs (variable *within B. bifarius* SNP set) using the SplitsTree neighbor‐net algorithm, uncorrected *P*‐distances, and heterozygous/ambiguous site states averaged.
**Appendix S6.** RNAseq smartpca analysis using only synonymous sites separated by >20 kb, with no missing genotype data (*N* = 1568 SNPs).
**Appendix S7.** (A) Mean estimated log natural probability (y‐axis 1) and ∆*K* (y‐axis 2) for each putative number of clusters (*x*‐axis) tested by STRUCTURE and implemented using STRUCTURE Harvester. (B) STRUCTURE clustering analysis for *nearcticus*‐W and *nearcticus*‐C treated as an independent data set (i.e., missing data, polymorphic loci, and single SNP per RAD locus were determined and used for filtering from a data set that contained only the *B. b. nearcticus* specimens, rather than simply removing specimens from the existing STRUCTURE input created after filtering the entire data set) for *K* = 2 (*N* = 6572 SNPs).
**Appendix S8.** (A) Schematic of the three population isolation with migration model used for the demographic analysis with *∂a∂I,* and a table of the resulting scaled parameter estimates from optimization of the model, with contemporary migration parameters highlighted. (B) Plots of model‐data comparisons for RNAseq (upper four rows) and ddRAD (lower four rows) SNP data.
**Appendix S9.** Sliding window analysis identifying regions containing high differentiation between *bifarius*‐E and each *nearcticus* population for RNAseq‐derived SNPs (average *F*_ST_ ≥ 0.9) across *B. impatiens* scaffolds.
