# Supplementary material for: Population genomics of divergence among extreme and intermediate color forms in a polymorphic insect
Source: Ecol Evol. 2016 Jan 3;6(4):1075–91. doi: 10.1002/ece3.1928 (PMC4722823; doi:10.1002/ece3.1928)
Supplement: Supplementary file 1 — Appendix S1. RNA Illumina sequencing coverage for each sample used in analyses, including total numbers of reads (averaged of the two paired ends), percentage passing TrimGalore! quality control, and the percentage successfully mapped by Tophat with a mismatch setting = 3 per read. Appendix S2. (A) Distribution of sequencing coverage per SNP per individual for the 46,259 B. bifarius RNAseq SNPs, with a mean = 66× (69 SD) coverage per SNP per individual. (B) Distribution of sequencing coverage per SNP per individual for the 25,308 B. bifarius ddRAD SNPs, with a mean = 31× (9 SD) coverage per SNP per individual. Appendix S3. Illumina sequencing information for ddRAD samples used in analysis, with total number of reads and number and percentage of reads retained after quality control (QC) with Stacks process_radtags. Appendix S4. Examples of sensitivity of node ages for SNAPP clades explored under different α and β prior settings, showing changes in absolute node age, but stability of relative node ages. Appendix S5. Phlyogenetic network for 46,259 RNAseq SNPs (variable within B. bifarius SNP set) using the SplitsTree neighbor‐net algorithm, uncorrected P‐distances, and heterozygous/ambiguous site states averaged. Appendix S6. RNAseq smartpca analysis using only synonymous sites separated by >20 kb, with no missing genotype data (N = 1568 SNPs). Appendix S7. (A) Mean estimated log natural probability (y‐axis 1) and ∆K (y‐axis 2) for each putative number of clusters (x‐axis) tested by STRUCTURE and implemented using STRUCTURE Harvester. (B) STRUCTURE clustering analysis for nearcticus‐W and nearcticus‐C treated as an independent data set (i.e., missing data, polymorphic loci, and single SNP per RAD locus were determined and used for filtering from a data set that contained only the B. b. nearcticus specimens, rather than simply removing specimens from the existing STRUCTURE input created after filtering the entire data set) for K = 2 (N = 6572 SNPs). Appendix S8. (A) Sc [file ECE3-6-1075-s001.docx]

**Appendices**

**Appendix 1.** RNA Illumina sequencing coverage for each sample used in analyses, including total numbers of reads (averaged of the two paired ends), percentage passing TrimGalore! quality control, and the percentage successfully mapped by Tophat with a mismatch setting = 3 per read.

| Sample | Population | Average of Number Reads | Average % passing QC for Paired Ends* | Average % Mapping for QC’d Paired Ends* |
| --- | --- | --- | --- | --- |
| Bbif_JDL121 | *nearcticus*-W | 9233081 | 97.5% | 75.2% |
| Bbif_JDL129 |  | 9827844 | 96.7% | 79.6% |
| Bbif_JDL132 |  | 7498253 | 95.9% | 67.2% |
| Bbif_JDL133 |  | 8450740 | 97.6% | 80.5% |
| Bbif_JDL134 |  | 9073953 | 97.7% | 69.0% |
| Bbif_JDL140 |  | 8871265 | 97.3% | 66.6% |
| Bbif_JDL211 |  | 10467091 | 97.0% | 76.1% |
| Bbif_JDL354** | *nearcticus*-C | 7826342 | 97.1% | 82.5% |
| Bbif_JDL359 |  | 7537896 | 97.0% | 80.2% |
| Bbif_JDL362 |  | 8294251 | 96.5% | 77.1% |
| Bbif_JDL371 |  | 5788158 | 97.2% | 81.2% |
| Bbif_JDL376 |  | 10220734 | 97.5% | 73.2% |
| Bbif_JDL383 |  | 9103332 | 97.2% | 81.9% |
| Bbif_JDL385 |  | 8457766 | 97.0% | 79.9% |
| Bbif_JDL388 |  | 8447189 | 96.7% | 67.3% |
| Bbif_JDL414 |  | 8473527 | 97.0% | 79.9% |
| Bbif_JDL389 | *bifarius*-E | 7730056 | 97.2% | 61.6% |
| Bbif_JDL399 |  | 8301225 | 97.0% | 77.1% |
| Bbif_JDL404 |  | 9268836 | 97.1% | 81.1% |
| Bbif_JDL410 |  | 9486506 | 97.1% | 81.1% |
| Bbif_JDL411 |  | 8287020 | 96.9% | 82.1% |
| Bbif_JDL413 |  | 8635730 | 97.6% | 60.2% |
| Bvos_JDL284 | *B. vosnesenskii* | 9374484 | 96.6% | 80.6% |
| *Values presented represent average numbers for the paired end reads in each individual  ** Specimen Identified as probable sister to JDL359 based on unusually high pairwise relatedness (*r* > 0.5), versus most pairs of samples, where *r* was near 0 (unrelated) or 1 (for self); final analyses excluded JDL354 from variant calling | | | | |

**Appendix 2.** (A) Distribution of sequencing coverage per SNP per individual for the 46,259 *B. bifarius* RNAseq SNPs, with a mean = 66X (69 SD) coverage per SNP per individual. (B) Distribution of sequencing coverage per SNP per individual for the 25,308 *B. bifarius* ddRAD SNPs, with a mean = 31X (9 SD) coverage per SNP per individual.

**Appendix 3:** Illumina sequencing information for ddRAD samples used in analysis, with total number of reads and number and percentage of reads retained after quality control (QC) with Stacks *process_radtags*.

| Sample | Population | Total number of reads | Number of reads retained after QC | Percent of reads retained |
| --- | --- | --- | --- | --- |
| JDL112 | *nearcticus*-W | 372569 | 303526 | 81.47% |
| JDL113^1^ |  | 389020 | 286994 | 73.77% |
| JDL114 |  | 452135 | 419077 | 92.69% |
| JDL117 |  | 571443 | 544070 | 95.21% |
| JDL119^1^ |  | 536600 | 475909 | 88.69% |
| JDL122 |  | 557755 | 507677 | 91.02% |
| JDL123 |  | 455432 | 380500 | 83.55% |
| JDL126 |  | 493920 | 451200 | 91.35% |
| JDL127 |  | 474323 | 405336 | 85.46% |
| JDL132 |  | 517713 | 460795 | 89.01% |
| JDL133 |  | 786039 | 707115 | 89.96% |
| JDL134^2^ |  | 641756 | 584855 | 91.13% |
| JDL135 |  | 666997 | 608943 | 91.30% |
| JDL136 |  | 664208 | 614206 | 92.47% |
| JDL138 |  | 733798 | 681534 | 92.88% |
| JDL139 |  | 777167 | 710050 | 91.36% |
| JDL141^2^ |  | 535218 | 480746 | 89.82% |
| JDL211 |  | 1011502 | 931643 | 92.10% |
| JDL348 | *nearcticus-C* | 782120 | 694700 | 88.82% |
| JDL357 |  | 718269 | 642184 | 89.41% |
| JDL358^3^ |  | 886945 | 826977 | 93.24% |
| JDL361 |  | 539348 | 513220 | 95.16% |
| JDL362 |  | 816481 | 757312 | 92.75% |
| JDL363 |  | 576746 | 534772 | 92.72% |
| JDL370^3^ |  | 668953 | 620383 | 92.74% |
| JDL372 |  | 570157 | 514398 | 90.22% |
| JDL373 |  | 425183 | 388262 | 91.32% |
| JDL374 |  | 521382 | 489156 | 93.82% |
| JDL378 |  | 397925 | 360591 | 90.62% |
| JDL379 |  | 438994 | 385682 | 87.86% |
| JDL381^4^ |  | 649436 | 590984 | 91.00% |
| JDL387^4^ |  | 634422 | 589145 | 92.86% |
| JDL388 |  | 742757 | 674754 | 90.84% |
| JDL414 |  | 316686 | 274521 | 86.69% |
| JDL389 | *bifarius-E* | 723566 | 661179 | 91.38% |
| JDL391 |  | 724624 | 676602 | 93.37% |
| JDL393 |  | 678675 | 620518 | 91.43% |
| JDL395^5^ |  | 458244 | 416730 | 90.94% |
| JDL397 |  | 549580 | 501552 | 91.26% |
| JDL401 |  | 798803 | 737985 | 92.39% |
| JDL403^5^ |  | 518310 | 487243 | 94.01% |
| JDL405 |  | 549126 | 496749 | 90.46% |
| JDL406 |  | 851111 | 786317 | 92.39% |
| JDL408 |  | 448959 | 420042 | 93.56% |
| JDL410* |  | 160055 | 125818 | 78.61% |
| JDL411 |  | 638459 | 576847 | 90.35% |
| JDL413 |  | 392277 | 351434 | 89.59% |

^1^Superscript numbers reflect potential sibling pairs identified by pairwise relatedness. In each case, the individual with the largest number of reads retained after quality control was kept for analyses.

*Individual excluded due to the small number of reads.

**Appendix 4:** Examples of sensitivity of node ages for SNAPP clades explored under different *α* and *β* prior settings, showing changes in absolute node age, but stability of relative node ages.

|  | **Node Ages For Different Priors** | | | **Relative Node Ages For Different Priors** | | |
| --- | --- | --- | --- | --- | --- | --- |
|  | **Default** | **α=1/β=200** | **α=10/β=200** | **Default** | **α=1/β=200** | **α=10/β=200** |
| *impatiens* | 2.62E-02 | 9.28E-03 | 2.19E-02 |  |  |  |
| *vosnesenskii* | 1.52E-02 | 4.36E-03 | 1.30E-02 | 5.82E-01 | 4.70E-01 | 5.96E-01 |
| *bifarius* | 5.09E-03 | 1.44E-03 | 4.18E-03 | 3.34E-01 | 3.30E-01 | 3.21E-01 |
| *nearcticus* | 5.64E-04 | 1.64E-04 | 4.83E-04 | 1.11E-01 | 1.14E-01 | 1.16E-01 |
| Node ages are presented in default scaled coalescent units. Relative node ages presented represent the age of the node of a given row divided by the age of the next oldest node in the row above. All SNAPP results from different analyses produced the same topology as Fig 2B with 100% support for each clade. | | | | | | |

**Appendix 5.** Phlyogenetic network for 46,259 RNAseq SNPs (variable *within B. bifarius* SNP set) using the SplitsTree neighbor-net algorithm, uncorrected *P*-distances, and heterozygous/ambiguous site states averaged. See Figure 2C of main text for comparison to ddRAD network.


**Appendix 6.** RNAseq smartpca analysis using only synonymous sites separated by >20kb, with no missing genotype data (N=1,568 SNPs). See Fig 3 in the main text for the full SNP set PCA.

**Appendix 7.** (A) Mean estimated log natural probability (y-axis 1) and ∆*K* (y-axis 2) for each putative number of clusters (x-axis) tested by STRUCTURE and implemented using STRUCTURE Harvester. Note ∆*K* values are not possible for *K* = 1 or for the last *K* value. (B) STRUCTURE clustering analysis for *nearcticus*-W and *nearcticus*-C treated as an independent data set (i.e., missing data, polymorphic loci, and single SNP per RAD locus were determined and used for filtering from a data set that contained only the *B. b. nearcticus* specimens, rather than simply removing specimens from the existing STRUCTURE input created after filtering the entire data set) for *K* = 2 (N = 6,572 SNPs)

**Appendix 8.** (A) Schematic of the three population isolation with migration model used for the demographic analysis with *∂a∂I,* and a table of the resulting scaled parameter estimates from optimization of the model, with contemporary migration parameters highlighted. Parameters were checked for similarity across runs from different optimization runs using different start values; some small differences were observed with different start parameters but overall were very similar within and between data sets and would not alter any conclusions. Model and optimization code are available on DRYAD. (B) Plots of model-data comparisons for RNAseq (upper four rows) and ddRAD (lower four rows) SNP data. In both RNAseq and ddRAD data sets, the complete SNP sets were supplied as inputs (e.g., no MAF or linkage filtering; linkage does not bias parameter estimation) but were projected down to 10 and 16 sampled alleles per population, respectively. For each data set the top row shows the pairwise two-dimensional folded site frequency spectrum from the data and the second row shows that estimated from the model. The third row shows the residuals between the model and data, with residual values plotted as histograms in the fourth row. Note the greater model-data similarity for the ddRAD data for the *nearcticus-bifarius* comparisons, which may further support the possible contribution from non-neutral evolution in gene-coding regions discussed with relation to *F*_ST_ comparisons (Table 2 of main text).

**Appendix 9.** Sliding window analysis identifying regions containing high differentiation between *bifarius*-E and each *nearcticus* population for RNAseq-derived SNPs (average *F*_ST_ ≥ 0.9) across *B. impatiens* scaffolds. Non-overlapping sliding window and *F*_ST_ calculation performed with vcftools (Danacek et al. 2011). For both pairs, an average of 3.2 SNPs per window was examined (range: 1-14). No high-divergence windows were identified between *nearcticus* populations.

| *bifarius-*E vs *nearcticus-*W | | | | *bifarius-*E vs *nearcticus-C* | | | |
| --- | --- | --- | --- | --- | --- | --- | --- |
| *B. imp* scaffold | WindowStart | No. SNPs | Mean *F*_ST_ | *B. imp* scaffold | WindowStart | No. SNPs | Mean *F*_ST_ |
| NT_176427.1 | 20001 | 1 | 1.00 | NT_176427.1 | 20001 | 1 | 1.00 |
| NT_176427.1 | 40001 | 2 | 1.00 | NT_176427.1 | 40001 | 2 | 1.00 |
| NT_176427.1 | 200001 | 2 | 1.00 | NT_176427.1 | 200001 | 2 | 1.00 |
| NT_176430.1 | 480001 | 2 | 1.00 | NT_176430.1 | 480001 | 2 | 1.00 |
| NT_176430.1 | 620001 | 3 | 1.00 | NT_176430.1 | 620001 | 4 | 0.95 |
| NT_176432.1 | 100001 | 1 | 1.00 | NT_176432.1 | 80001 | 1 | 0.92 |
| NT_176438.1 | 140001 | 4 | 0.96 | NT_176432.1 | 100001 | 1 | 1.00 |
| NT_176438.1 | 1640001 | 1 | 1.00 | NT_176432.1 | 120001 | 5 | 0.91 |
| NT_176438.1 | 1720001 | 1 | 1.00 | NT_176436.1 | 360001 | 14 | 0.90 |
| NT_176439.1 | 320001 | 1 | 1.00 | NT_176438.1 | 140001 | 5 | 0.92 |
| NT_176442.1 | 180001 | 1 | 1.00 | NT_176438.1 | 1640001 | 1 | 1.00 |
| NT_176446.1 | 1520001 | 6 | 0.90 | NT_176438.1 | 1720001 | 1 | 1.00 |
| NT_176452.1 | 1640001 | 6 | 0.94 | NT_176439.1 | 320001 | 1 | 1.00 |
| NT_176455.1 | 40001 | 1 | 1.00 | NT_176452.1 | 1640001 | 6 | 0.92 |
| NT_176455.1 | 120001 | 9 | 0.92 | NT_176455.1 | 40001 | 1 | 1.00 |
| NT_176463.1 | 1460001 | 4 | 0.95 | NT_176455.1 | 120001 | 9 | 0.93 |
| NT_176468.1 | 720001 | 1 | 1.00 | NT_176463.1 | 1460001 | 3 | 0.95 |
| NT_176479.1 | 100001 | 1 | 1.00 | NT_176468.1 | 720001 | 1 | 1.00 |
| NT_176494.1 | 100001 | 1 | 1.00 | NT_176468.1 | 880001 | 3 | 0.90 |
| NT_176499.1 | 200001 | 3 | 1.00 | NT_176479.1 | 100001 | 1 | 1.00 |
| NT_176499.1 | 220001 | 3 | 1.00 | NT_176494.1 | 100001 | 1 | 1.00 |
| NT_176499.1 | 260001 | 2 | 1.00 | NT_176499.1 | 200001 | 3 | 1.00 |
| NT_176499.1 | 1200001 | 9 | 0.94 | NT_176499.1 | 220001 | 3 | 1.00 |
| NT_176510.1 | 340001 | 1 | 0.92 | NT_176499.1 | 260001 | 2 | 1.00 |
| NT_176514.1 | 780001 | 3 | 0.90 | NT_176499.1 | 1200001 | 10 | 0.91 |
| NT_176514.1 | 860001 | 2 | 1.00 | NT_176510.1 | 340001 | 1 | 0.92 |
| NT_176518.1 | 1280001 | 2 | 1.00 | NT_176514.1 | 780001 | 3 | 0.93 |
| NT_176525.1 | 580001 | 1 | 1.00 | NT_176514.1 | 860001 | 2 | 1.00 |
| NT_176532.1 | 20001 | 2 | 0.92 | NT_176518.1 | 1280001 | 2 | 1.00 |
| NT_176532.1 | 480001 | 13 | 0.94 | NT_176532.1 | 480001 | 12 | 0.96 |
| NT_176533.1 | 660001 | 5 | 0.95 | NT_176533.1 | 1100001 | 1 | 0.91 |
| NT_176533.1 | 1100001 | 1 | 0.91 | NT_176533.1 | 1520001 | 1 | 1.00 |
| NT_176533.1 | 1520001 | 1 | 1.00 | NT_176537.1 | 1 | 1 | 1.00 |
| NT_176537.1 | 1 | 1 | 1.00 | NT_176539.1 | 1 | 1 | 1.00 |
| NT_176539.1 | 1 | 1 | 1.00 | NT_176548.1 | 20001 | 3 | 0.92 |
| NT_176548.1 | 1460001 | 3 | 1.00 | NT_176548.1 | 1160001 | 2 | 0.92 |
| NT_176554.1 | 80001 | 10 | 0.91 | NT_176548.1 | 1460001 | 4 | 0.94 |
| NT_176554.1 | 2600001 | 4 | 0.95 | NT_176554.1 | 80001 | 11 | 0.90 |
| NT_176561.1 | 2740001 | 5 | 0.97 | NT_176554.1 | 2600001 | 4 | 0.96 |
| NT_176563.1 | 560001 | 5 | 0.90 | NT_176561.1 | 2740001 | 5 | 0.95 |
| NT_176565.1 | 560001 | 1 | 1.00 | NT_176563.1 | 560001 | 5 | 0.92 |
| NT_176574.1 | 640001 | 11 | 1.00 | NT_176565.1 | 560001 | 1 | 1.00 |
| NT_176574.1 | 660001 | 4 | 1.00 | NT_176574.1 | 640001 | 11 | 1.00 |
| NT_176574.1 | 680001 | 5 | 0.94 | NT_176574.1 | 660001 | 4 | 0.97 |
| NT_176574.1 | 740001 | 5 | 0.98 | NT_176574.1 | 680001 | 5 | 0.91 |
| NT_176574.1 | 760001 | 9 | 1.00 | NT_176574.1 | 740001 | 5 | 0.99 |
| NT_176574.1 | 780001 | 1 | 1.00 | NT_176574.1 | 760001 | 9 | 1.00 |
| NT_176574.1 | 1440001 | 2 | 1.00 | NT_176574.1 | 780001 | 1 | 1.00 |
| NT_176610.1 | 200001 | 4 | 0.93 | NT_176574.1 | 1440001 | 2 | 1.00 |
| NT_176616.1 | 1440001 | 1 | 0.92 | NT_176593.1 | 140001 | 5 | 0.93 |
| NT_176622.1 | 100001 | 1 | 1.00 | NT_176610.1 | 200001 | 4 | 0.94 |
| NT_176622.1 | 120001 | 4 | 0.90 | NT_176622.1 | 300001 | 5 | 1.00 |
| NT_176622.1 | 300001 | 7 | 0.91 | NT_176622.1 | 520001 | 5 | 0.96 |
| NT_176622.1 | 520001 | 5 | 0.97 | NT_176622.1 | 920001 | 3 | 0.93 |
| NT_176622.1 | 1040001 | 2 | 0.93 | NT_176622.1 | 1580001 | 1 | 1.00 |
| NT_176622.1 | 1320001 | 4 | 0.94 | NT_176622.1 | 1600001 | 3 | 0.94 |
| NT_176622.1 | 1580001 | 1 | 1.00 | NT_176622.1 | 1620001 | 1 | 1.00 |
| NT_176622.1 | 1620001 | 1 | 1.00 | NT_176636.1 | 1040001 | 1 | 1.00 |
| NT_176636.1 | 1040001 | 1 | 1.00 | NT_176636.1 | 1260001 | 3 | 0.92 |
| NT_176636.1 | 1260001 | 3 | 0.96 | NT_176638.1 | 240001 | 1 | 1.00 |
| NT_176636.1 | 1280001 | 2 | 0.93 | NT_176638.1 | 740001 | 10 | 0.91 |
| NT_176638.1 | 740001 | 9 | 0.91 | NT_176644.1 | 1620001 | 3 | 0.90 |
| NT_176644.1 | 1620001 | 3 | 0.91 | NT_176652.1 | 180001 | 1 | 1.00 |
| NT_176652.1 | 180001 | 1 | 1.00 | NT_176656.1 | 1 | 3 | 1.00 |
| NT_176652.1 | 280001 | 3 | 0.90 | NT_176656.1 | 160001 | 1 | 1.00 |
| NT_176656.1 | 1 | 3 | 1.00 | NT_176667.1 | 1200001 | 3 | 0.94 |
| NT_176656.1 | 160001 | 1 | 1.00 | NT_176667.1 | 1600001 | 2 | 0.95 |
| NT_176667.1 | 1200001 | 3 | 0.96 | NT_176678.1 | 100001 | 3 | 0.97 |
| NT_176667.1 | 1600001 | 2 | 0.96 | NT_176678.1 | 360001 | 4 | 0.96 |
| NT_176678.1 | 100001 | 3 | 0.93 | NT_176715.1 | 380001 | 4 | 0.95 |
| NT_176678.1 | 360001 | 5 | 0.90 | NT_176717.1 | 240001 | 4 | 0.97 |
| NT_176683.1 | 980001 | 10 | 0.90 | NT_176736.1 | 740001 | 7 | 0.92 |
| NT_176683.1 | 1000001 | 2 | 0.92 | NT_176736.1 | 900001 | 6 | 0.91 |
| NT_176715.1 | 380001 | 4 | 0.94 | NT_176737.1 | 1840001 | 4 | 0.93 |
| NT_176717.1 | 240001 | 4 | 0.93 | NT_176737.1 | 1940001 | 9 | 0.93 |
| NT_176736.1 | 40001 | 3 | 0.92 | NT_176737.1 | 2020001 | 1 | 1.00 |
| NT_176736.1 | 740001 | 7 | 0.96 | NT_176737.1 | 2240001 | 6 | 1.00 |
| NT_176736.1 | 900001 | 6 | 0.92 | NT_176737.1 | 2320001 | 4 | 0.94 |
| NT_176737.1 | 1840001 | 4 | 0.92 | NT_176739.1 | 240001 | 2 | 0.91 |
| NT_176737.1 | 2020001 | 1 | 1.00 | NT_176739.1 | 460001 | 1 | 1.00 |
| NT_176737.1 | 2240001 | 6 | 0.98 | NT_176739.1 | 1060001 | 3 | 0.93 |
| NT_176737.1 | 2320001 | 4 | 0.93 | NT_176739.1 | 1100001 | 1 | 0.92 |
| NT_176739.1 | 460001 | 1 | 1.00 | NT_176739.1 | 1120001 | 8 | 0.97 |
| NT_176739.1 | 1100001 | 1 | 1.00 | NT_176739.1 | 1140001 | 4 | 0.93 |
| NT_176739.1 | 1120001 | 8 | 0.97 | NT_176739.1 | 1180001 | 8 | 0.96 |
| NT_176739.1 | 1140001 | 3 | 1.00 | NT_176751.1 | 100001 | 2 | 1.00 |
| NT_176739.1 | 1180001 | 8 | 0.93 | NT_176751.1 | 240001 | 1 | 1.00 |
| NT_176744.1 | 20001 | 1 | 1.00 | NT_176751.1 | 320001 | 5 | 1.00 |
| NT_176751.1 | 100001 | 2 | 1.00 | NT_176779.1 | 260001 | 1 | 1.00 |
| NT_176751.1 | 160001 | 10 | 0.93 | NT_176780.1 | 140001 | 6 | 0.93 |
| NT_176751.1 | 180001 | 7 | 0.90 | NT_176781.1 | 80001 | 2 | 1.00 |
| NT_176751.1 | 240001 | 1 | 1.00 | NT_176781.1 | 140001 | 2 | 1.00 |
| NT_176751.1 | 320001 | 5 | 1.00 | NT_176796.1 | 60001 | 3 | 0.97 |
| NT_176779.1 | 260001 | 1 | 1.00 | NT_176796.1 | 480001 | 4 | 0.95 |
| NT_176781.1 | 80001 | 2 | 1.00 | NT_176796.1 | 580001 | 1 | 0.92 |
| NT_176781.1 | 140001 | 2 | 1.00 | NT_176796.1 | 620001 | 4 | 0.94 |
| NT_176796.1 | 60001 | 3 | 0.93 | NT_176796.1 | 640001 | 2 | 1.00 |
| NT_176796.1 | 480001 | 4 | 0.97 | NT_176808.1 | 2320001 | 1 | 1.00 |
| NT_176796.1 | 520001 | 4 | 0.97 | NT_176822.1 | 160001 | 4 | 0.94 |
| NT_176796.1 | 580001 | 1 | 0.92 | NT_176837.1 | 520001 | 1 | 1.00 |
| NT_176796.1 | 620001 | 4 | 0.94 | NT_176837.1 | 2720001 | 1 | 0.92 |
| NT_176796.1 | 640001 | 2 | 1.00 | NT_176837.1 | 3680001 | 1 | 1.00 |
| NT_176808.1 | 2320001 | 1 | 1.00 | NT_176861.1 | 1420001 | 2 | 0.93 |
| NT_176822.1 | 160001 | 3 | 1.00 | NT_176871.1 | 760001 | 1 | 1.00 |
| NT_176837.1 | 520001 | 1 | 1.00 | NT_176882.1 | 220001 | 1 | 1.00 |
| NT_176837.1 | 2720001 | 1 | 0.92 | NT_176882.1 | 2220001 | 2 | 1.00 |
| NT_176837.1 | 3680001 | 1 | 0.92 | NT_176893.1 | 40001 | 6 | 0.90 |
| NT_176855.1 | 60001 | 2 | 0.92 | NT_176897.1 | 120001 | 1 | 0.92 |
| NT_176861.1 | 1420001 | 2 | 0.92 | NT_176897.1 | 620001 | 2 | 1.00 |
| NT_176871.1 | 760001 | 1 | 1.00 | NT_176897.1 | 1600001 | 7 | 0.95 |
| NT_176882.1 | 220001 | 1 | 1.00 | NT_176897.1 | 1880001 | 4 | 0.91 |
| NT_176882.1 | 2220001 | 2 | 1.00 | NT_176897.1 | 2120001 | 1 | 1.00 |
| NT_176885.1 | 340001 | 1 | 1.00 | NT_176906.1 | 20001 | 2 | 1.00 |
| NT_176897.1 | 120001 | 1 | 1.00 | NT_176906.1 | 100001 | 6 | 0.97 |
| NT_176897.1 | 620001 | 3 | 0.93 | NT_176962.1 | 2100001 | 4 | 0.94 |
| NT_176897.1 | 1600001 | 7 | 0.96 | NT_176967.1 | 1060001 | 2 | 1.00 |
| NT_176897.1 | 2120001 | 1 | 1.00 | NT_176984.1 | 20001 | 2 | 1.00 |
| NT_176906.1 | 20001 | 2 | 1.00 | NT_177000.1 | 540001 | 1 | 1.00 |
| NT_176906.1 | 100001 | 5 | 1.00 | NT_177001.1 | 240001 | 2 | 0.96 |
| NT_176962.1 | 2100001 | 4 | 0.92 | NT_177001.1 | 860001 | 1 | 1.00 |
| NT_176967.1 | 920001 | 2 | 0.92 | NT_177001.1 | 1040001 | 1 | 1.00 |
| NT_176967.1 | 1060001 | 2 | 1.00 | NT_177001.1 | 1420001 | 6 | 0.95 |
| NT_176984.1 | 20001 | 2 | 1.00 | NT_177030.1 | 20001 | 5 | 0.95 |
| NT_177000.1 | 420001 | 6 | 0.94 | NT_177040.1 | 40001 | 9 | 0.93 |
| NT_177001.1 | 240001 | 2 | 1.00 | NT_177056.1 | 120001 | 8 | 0.98 |
| NT_177001.1 | 860001 | 1 | 1.00 | NT_177059.1 | 1240001 | 5 | 1.00 |
| NT_177001.1 | 1040001 | 1 | 1.00 | NT_177064.1 | 200001 | 1 | 1.00 |
| NT_177001.1 | 1420001 | 6 | 0.94 | NT_177066.1 | 780001 | 1 | 1.00 |
| NT_177030.1 | 20001 | 5 | 0.94 | NT_177066.1 | 1840001 | 1 | 0.91 |
| NT_177040.1 | 240001 | 3 | 0.96 | NT_177083.1 | 380001 | 7 | 1.00 |
| NT_177056.1 | 120001 | 7 | 1.00 | NT_177094.1 | 100001 | 8 | 0.96 |
| NT_177059.1 | 1240001 | 5 | 1.00 | NT_177126.1 | 20001 | 2 | 0.93 |
| NT_177064.1 | 200001 | 1 | 1.00 | NT_177126.1 | 40001 | 3 | 0.98 |
| NT_177066.1 | 440001 | 2 | 0.93 | NT_177143.1 | 1 | 1 | 0.92 |
| NT_177066.1 | 780001 | 1 | 1.00 | NT_177143.1 | 340001 | 5 | 0.91 |
| NT_177066.1 | 1880001 | 3 | 0.97 | NT_177154.1 | 20001 | 9 | 0.98 |
| NT_177083.1 | 380001 | 7 | 1.00 | NT_177154.1 | 40001 | 3 | 0.97 |
| NT_177094.1 | 100001 | 8 | 0.96 | NT_177154.1 | 160001 | 4 | 1.00 |
| NT_177126.1 | 20001 | 2 | 0.93 | NT_177158.1 | 80001 | 1 | 1.00 |
| NT_177126.1 | 40001 | 3 | 0.95 | NT_177194.1 | 1 | 3 | 1.00 |
| NT_177154.1 | 20001 | 8 | 1.00 | NT_177234.1 | 20001 | 1 | 1.00 |
| NT_177154.1 | 40001 | 3 | 0.97 | NT_177249.1 | 300001 | 3 | 0.94 |
| NT_177154.1 | 160001 | 4 | 0.98 | NT_177262.1 | 20001 | 1 | 1.00 |
| NT_177158.1 | 80001 | 1 | 1.00 | NT_177312.1 | 40001 | 1 | 1.00 |
| NT_177184.1 | 360001 | 2 | 0.93 | NT_177333.1 | 20001 | 1 | 1.00 |
| NT_177194.1 | 1 | 3 | 1.00 | NT_177333.1 | 280001 | 1 | 1.00 |
| NT_177234.1 | 20001 | 1 | 1.00 | NT_177333.1 | 600001 | 2 | 1.00 |
| NT_177249.1 | 140001 | 4 | 0.94 | NT_177333.1 | 740001 | 1 | 1.00 |
| NT_177249.1 | 300001 | 3 | 0.96 | NT_177474.1 | 1120001 | 1 | 1.00 |
| NT_177262.1 | 20001 | 1 | 1.00 | NT_177498.1 | 300001 | 9 | 0.93 |
| NT_177312.1 | 40001 | 1 | 1.00 | NT_177498.1 | 460001 | 6 | 0.92 |
| NT_177333.1 | 20001 | 1 | 0.92 | NT_177498.1 | 520001 | 4 | 0.95 |
| NT_177333.1 | 280001 | 1 | 1.00 | NT_177498.1 | 640001 | 2 | 1.00 |
| NT_177333.1 | 600001 | 2 | 1.00 | NT_177498.1 | 660001 | 5 | 0.94 |
| NT_177333.1 | 740001 | 1 | 1.00 | NT_177498.1 | 740001 | 6 | 0.91 |
| NT_177349.1 | 80001 | 3 | 0.92 | NT_177500.1 | 40001 | 1 | 1.00 |
| NT_177474.1 | 1120001 | 1 | 1.00 | NT_177563.1 | 1 | 2 | 0.96 |
| NT_177498.1 | 20001 | 1 | 0.92 | NT_177578.1 | 80001 | 5 | 0.98 |
| NT_177498.1 | 300001 | 10 | 0.93 | NT_177602.1 | 180001 | 1 | 0.92 |
| NT_177498.1 | 460001 | 6 | 0.92 | NT_177602.1 | 240001 | 1 | 1.00 |
| NT_177498.1 | 520001 | 4 | 0.94 | NT_177632.1 | 60001 | 11 | 1.00 |
| NT_177498.1 | 640001 | 2 | 1.00 | NT_177632.1 | 100001 | 1 | 0.92 |
| NT_177498.1 | 660001 | 4 | 1.00 | NT_177693.1 | 40001 | 1 | 1.00 |
| NT_177498.1 | 740001 | 5 | 0.95 | NT_177694.1 | 340001 | 2 | 1.00 |
| NT_177500.1 | 40001 | 1 | 1.00 | NT_177694.1 | 480001 | 3 | 0.91 |
| NT_177578.1 | 80001 | 5 | 0.98 | NT_177694.1 | 500001 | 3 | 1.00 |
| NT_177602.1 | 180001 | 1 | 1.00 | NT_177694.1 | 520001 | 1 | 1.00 |
| NT_177602.1 | 240001 | 1 | 1.00 | NT_177694.1 | 880001 | 3 | 0.98 |
| NT_177632.1 | 60001 | 11 | 1.00 | NT_177694.1 | 920001 | 3 | 0.94 |
| NT_177632.1 | 100001 | 1 | 1.00 | NT_177694.1 | 1200001 | 5 | 0.96 |
| NT_177693.1 | 40001 | 1 | 1.00 | NT_177753.1 | 60001 | 2 | 1.00 |
| NT_177694.1 | 340001 | 2 | 1.00 | NT_177755.1 | 1 | 1 | 1.00 |
| NT_177694.1 | 500001 | 3 | 1.00 | NT_177755.1 | 100001 | 1 | 1.00 |
| NT_177694.1 | 520001 | 1 | 1.00 | NT_177755.1 | 120001 | 1 | 1.00 |
| NT_177694.1 | 880001 | 3 | 0.97 | NT_177864.1 | 840001 | 4 | 0.94 |
| NT_177694.1 | 920001 | 3 | 0.93 | NT_178075.1 | 1 | 1 | 1.00 |
| NT_177694.1 | 1200001 | 5 | 0.95 | NT_178126.1 | 360001 | 1 | 1.00 |
| NT_177753.1 | 60001 | 2 | 0.96 | NT_178930.1 | 1 | 1 | 1.00 |
| NT_177753.1 | 80001 | 5 | 0.91 | NT_179184.1 | 1 | 2 | 0.93 |
| NT_177753.1 | 140001 | 11 | 0.90 | NT_179893.1 | 1400001 | 2 | 1.00 |
| NT_177755.1 | 1 | 1 | 1.00 | NT_180140.1 | 200001 | 8 | 0.98 |
| NT_177755.1 | 100001 | 1 | 1.00 | NT_180140.1 | 220001 | 1 | 1.00 |
| NT_177755.1 | 120001 | 1 | 1.00 | NT_180431.1 | 200001 | 1 | 1.00 |
| NT_177864.1 | 840001 | 4 | 0.94 | NT_180435.1 | 560001 | 2 | 0.96 |
| NT_178075.1 | 1 | 1 | 1.00 | NT_180435.1 | 580001 | 1 | 1.00 |
| NT_178126.1 | 360001 | 1 | 1.00 |  |  |  |  |
| NT_178930.1 | 1 | 1 | 1.00 |  |  |  |  |
| NT_179893.1 | 580001 | 6 | 0.97 |  |  |  |  |
| NT_179893.1 | 1400001 | 2 | 1.00 |  |  |  |  |
| NT_180140.1 | 200001 | 8 | 0.98 |  |  |  |  |
| NT_180140.1 | 220001 | 1 | 1.00 |  |  |  |  |
| NT_180431.1 | 200001 | 1 | 1.00 |  |  |  |  |
| NT_180435.1 | 580001 | 1 | 1.00 |  |  |  |  |
| NT_180435.1 | 820001 | 1 | 1.00 |  |  |  |  |
